# Rare Cutaneous Presentation of Disseminated Cryptococcus gattii Infection in an Immunocompetent Male: A Case Report

**DOI:** 10.7759/cureus.100079

**Published:** 2025-12-25

**Authors:** Sofia De La O Villalobos, Jesús Eduardo Adame-Garza, Mariana Gonzalez Plascencia, Adriana Saenz Ramirez, Raúl Isaí Castillo Cabrera, Ivan Omar Gómez Figueroa

**Affiliations:** 1 Internal Medicine, Autonomous University of Chihuahua, Hospital General "Presidente Lázaro Cárdenas", Chihuahua, MEX; 2 Internal Medicine/Infectious Diseases, El Instituto Nacional de Enfermedades Respiratorias Ismael Cosío Villegas, Mexico City, MEX; 3 Hematology, Centro Medico Nacional 20 de Noviembre/Universidad Nacional Autónoma de México, Mexico City, MEX; 4 Internal Medicine, Autonomous University of Chihuahua, Chihuahua, MEX

**Keywords:** cryptococcus gattii, cutaneous cryptococcosis, immunocompetent host, infectious dermatology, opportunistic mycosis

## Abstract

Cryptococcosis is a systemic mycosis caused by *Cryptococcus neoformans* and *Cryptococcus gattii*, affecting mainly immunocompromised and immunocompetent hosts, respectively. Cutaneous involvement may indicate secondary hematogenous dissemination from systemic disease or, less frequently, primary cutaneous cryptococcosis resulting from direct inoculation without systemic infection. Although cutaneous cryptococcosis can present with diverse morphologies, cutaneous involvement is uncommon in *C. gattii* infection and is more frequently associated with *C. neoformans*. We present the case of a 56-year-old immunocompetent man from northern Mexico who developed progressive neurological symptoms accompanied by disseminated cutaneous macules. Cerebrospinal fluid and skin biopsy confirmed *C. gattii*. The case highlights the need to consider this pathogen in immunocompetent hosts with unexplained meningoencephalitis. Induction therapy with liposomal amphotericin B and fluconazole was initiated, leading to resolution of the cutaneous manifestations.

## Introduction

Cryptococcosis is a systemic mycosis caused by *Cryptococcus neoformans *and *Cryptococcus gattii*. Because it primarily affects immunocompromised individuals, the *C. neoformans *species complex is regarded as an opportunistic pathogen. Cryptococcal infection is acquired via inhalation, followed by subsequent dissemination to the central nervous system to cause meningitis [1]. The *C. gattii *species complex, on the other hand, is considered a primary pathogen because it often infects people who seem to be immunocompetent. *C. gattii *species complex is acquired via environmental exposure, mainly by inhalation of spores from trees, soil, and decaying wood. It mostly affects the central nervous system and the respiratory system, both of which are associated with high morbidity and mortality [2].

Systemic manifestations may involve weight loss, fever, and chills. Neurological involvement can present with meningoencephalitis, headache, neck rigidity, fever, or changes in mental status. Pulmonary disease commonly manifests as cough, dyspnea, and chest pain [3]. *Cryptococcus *can cause a wide range of skin lesions, which frequently serve as an early indicator of disseminated disease. The initial lesions may appear as violaceous nodules, maculopapules with central ulceration, or papules [2]. The diagnosis of cryptococcosis requires a biopsy, a thorough epidemiological history, and the exclusion of underlying immunosuppression because there is no specific skin morphology. In our case, the immunocompetent patient presented with nonspecific skin lesions that did not initially suggest cryptococcosis; however, through a thorough clinical, microbiological, and epidemiological analysis, the diagnosis was guided toward *Cryptococcus* infection.

## Case presentation

A 56-year-old immunocompetent male, residing in a mountainous region of northern Mexico, with a significant history of chronic exposure to biomass combustion, particulate matter from a pasture mill, and continuous contact with farm animals, and with no known chronic comorbidities, presented with a one-month history of neurological symptoms. Symptoms began in early October, presenting with intense occipital headache accompanied by photophobia and recurrent emesis. On October 25, he experienced a generalized tonic-clonic seizure, prompting evaluation at multiple healthcare facilities where no definitive diagnosis was established. His clinical course subsequently progressed with persistent severe headache, spatial and temporal disorientation, and intermittent abnormal motor activity.

Cerebrospinal fluid analysis demonstrated a clear appearance, marked hyperproteinorrhachia (498 mg/dL), mildly decreased glucose, and predominant neutrophilic pleocytosis (98%). Potassium hydroxide (KOH) preparation and methylene blue stain were positive for fungal elements, and a FilmArray meningitis/encephalitis panel identified *Cryptococcus*; cerebrospinal fluid culture was positive for *C. gattii.*

**Figure 1:**
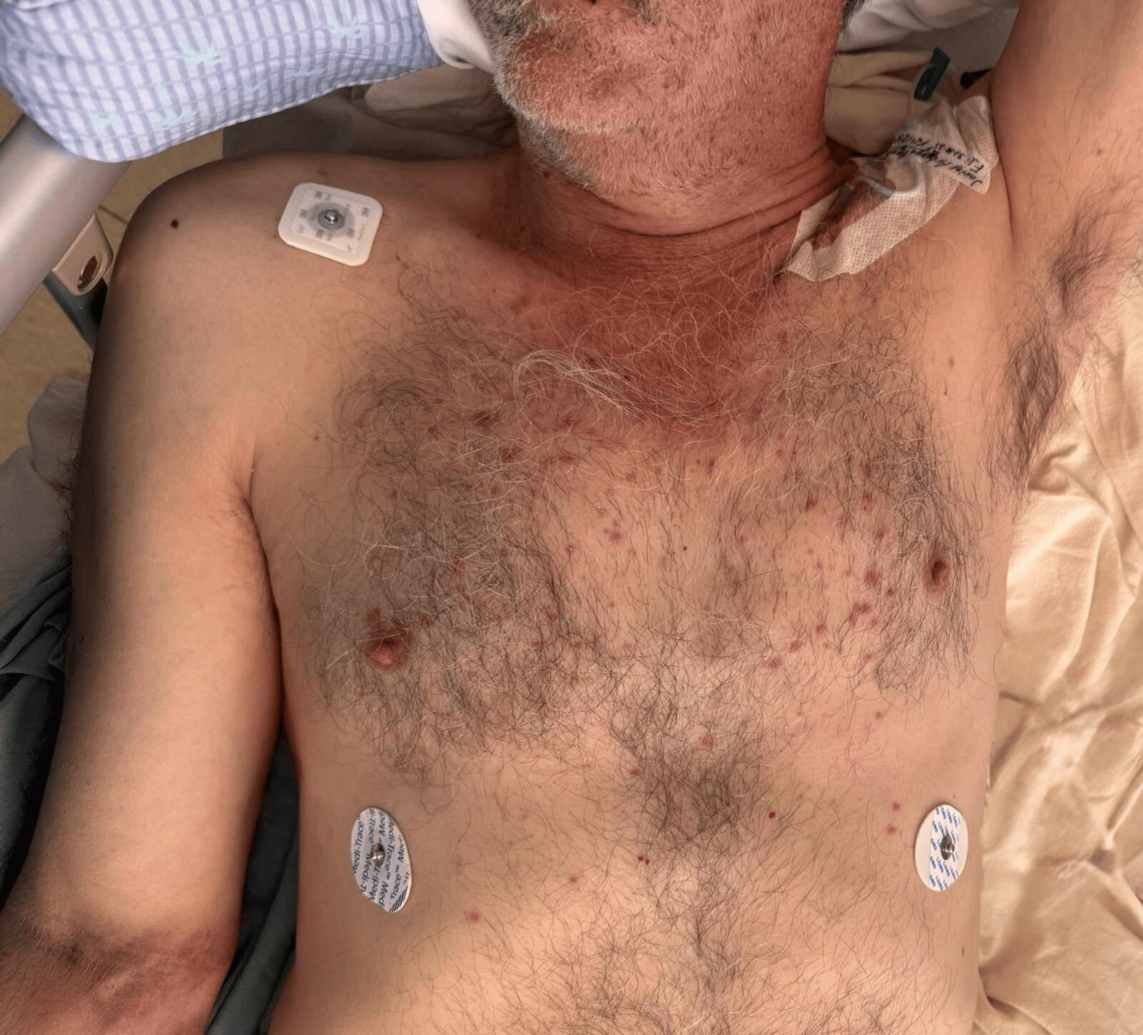
Disseminated dermatosis involving the trunk, characterized by numerous small, non-tender, millimetric macules over the chest.

**Figure 2:**
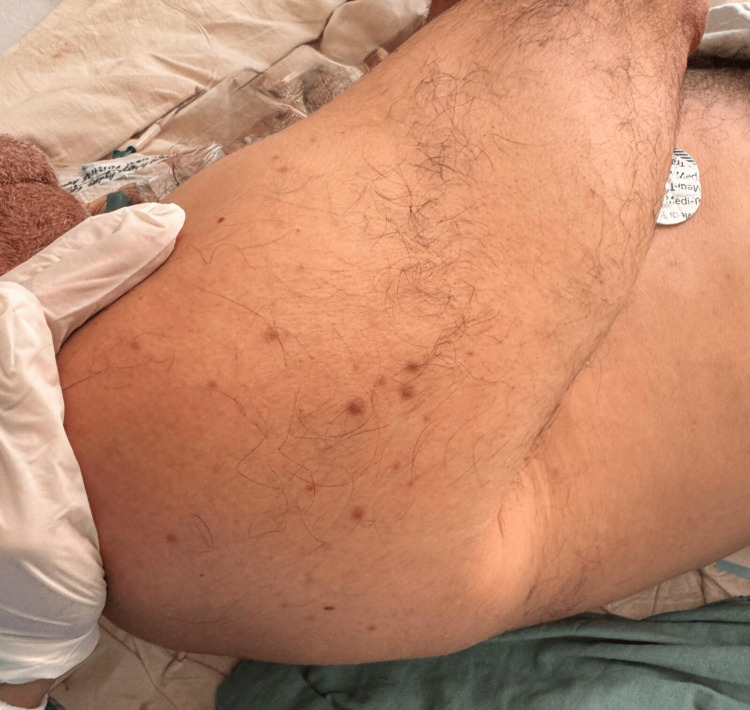
Disseminated dermatosis involving the right upper extremity at the shoulder, characterized by numerous small, non-tender, millimetric macules.

Dermatologic examination revealed a disseminated dermatosis involving the trunk (Figure 1), lower back, and right upper extremity (Figure 2), characterized by numerous small, non-tender, millimetric macules.

**Figure 3:**
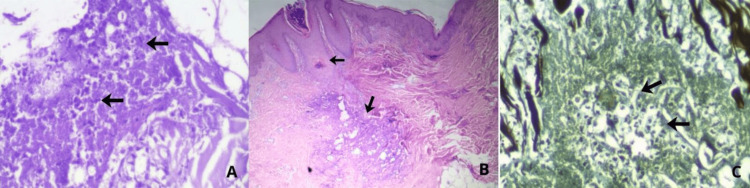
(A) Periodic acid-Schiff stain (PAS), 40×: Multiple round-to-oval yeast-like structures with narrow-based budding, each surrounded by a prominent clear halo. (B) Hematoxylin and eosin stain (H&E), 20×: Dense mixed inflammatory infiltrate in the dermis composed of lymphocytes, histiocytes, and neutrophils, with scattered round-to-oval yeast-like organisms. (C) Gomori-Grocott methenamine silver stain (GMS), 40×: Numerous yeast-like fungal structures sharply highlighted within the tissue.

Given the diagnostic suspicion, an incisional skin biopsy measuring 0.8 × 0.6 × 0.4 cm was performed. Histopathologic evaluation revealed a dense mixed inflammatory infiltrate within the dermis, composed of lymphocytes, histiocytes, and neutrophils. Interspersed throughout the infiltrate were numerous round-to-oval yeast-like structures exhibiting narrow-based budding and surrounded by a prominent clear halo, consistent with a polysaccharide capsule, findings morphologically compatible with *Cryptococcus *species. These characteristics were further supported by special stains: pPeriodic acid-Schiff stain (PAS) highlighting multiple encapsulated yeast-like organisms (Figure 3, Image A), hematoxylin and eosin stain (H&E) demonstrating the inflammatory background with scattered fungi (Figure 3, Image B), and Gomori-Grocott methenamine silver stain (GMS) sharply outlining numerous fungal structures within the tissue (Figure 3, Image C).

## Discussion

Cryptococcosis is a systemic opportunistic mycosis with worldwide distribution. It is caused by two distinct species, *C. neoformans *and *C. gattii. C. neoformans *predominantly affects immunocompromised individuals, whereas *C. gattii *more commonly infects immunocompetent hosts [4].

Cutaneous cryptococcosis may occur as secondary involvement, in which cutaneous lesions arise from hematogenous dissemination of a systemic infection, or as primary cutaneous cryptococcosis, caused by transcutaneous inoculation in the absence of systemic disease.

Up to 15% of acquired immunodeficiency syndrome (AIDS) patients may develop cutaneous lesions from cryptococcosis, which most frequently manifests as involvement of the central nervous system. In such cases, the skin findings typically represent secondary lesions that serve as early indicators of underlying systemic dissemination [5]. *C. gattii*-induced cutaneous cryptococcosis is uncommon and can appear as the only clinical sign of a spreading illness or as an early symptom [6]. The cutaneous findings are diverse and may appear as ulcers, plaques, cellulitis-like areas, abscesses, acneiform eruptions, pustules, papules, vesicles, or nodular lesions.

The diagnosis of cutaneous cryptococcosis can be established through direct mycological examination using India ink or saline, in which *Cryptococcus *appears as a yeast measuring 4-8 μm in diameter surrounded by a mucoid capsule two to three times larger [7]. Culture can be done on Niger seed agar or Sabouraud dextrose agar, which further supports the diagnosis. A skin biopsy (punch or excisional) typically reveals epidermal hyperplasia, a sparse chronic inflammatory infiltrate with histiocytes and occasional giant cells, and large clear spaces within the dermis filled with numerous encapsulated yeasts. The capsule stains with mucicarmine and Alcian blue, while fungal elements can be visualized with PAS and Grocott-Gomori stains. A systemic evaluation is mandatory in all cases, including neurological assessment, cerebrospinal fluid analysis, imaging studies (CT of the brain, chest, and abdomen), and screening for human immunodeficiency virus (HIV) and human T-lymphotropic virus type 1 (HTLV-1) [5].

Therapy includes regimens based on amphotericin B, 5-flucytosine, and fluconazole. This patient received induction therapy with liposomal amphotericin B plus fluconazole for a duration of four weeks, achieving adequate clinical improvement and complete resolution of the cutaneous lesions [7]. He is currently undergoing consolidation therapy with fluconazole.

## Conclusions

*C. gattii*-induced cutaneous cryptococcosis is a rare clinical manifestation that can present in a variety of ways, from acneiform eruptions to ulcers, plaques, nodules, or lesions resembling cellulitis. A thorough clinical assessment is essential, particularly with regard to the patient’s occupational and environmental exposures, given the association of *C. gattii *with certain geographic and ecological niches. Equally important is the systematic evaluation for potential systemic involvement, especially dissemination to the central nervous system, which can happen in immunocompetent hosts and has a major impact on prognosis and therapeutic decisions. Early recognition, appropriate microbiological confirmation, and comprehensive staging of the disease remain crucial steps in optimizing outcomes.
